# Genome-Wide Association Studies on Chinese Wheat Cultivars Reveal a Novel *Fusarium* Crown Rot Resistance Quantitative Trait Locus on Chromosome 3BL

**DOI:** 10.3390/plants13060856

**Published:** 2024-03-15

**Authors:** Chuyuan Wang, Manli Sun, Peipei Zhang, Xiaopeng Ren, Shuqing Zhao, Mengyu Li, Zhuang Ren, Meng Yuan, Linfei Ma, Zihan Liu, Kaixuan Wang, Feng Chen, Zaifeng Li, Xiaodong Wang

**Affiliations:** 1 State Key Laboratory of North China Crop Improvement and Regulation, College of Plant Protection, Hebei Agricultural University, Baoding 071000, China; wcy896105360@163.com (C.W.); sunmanli@hebau.edu.cn (M.S.); zhangpeijiayouba@163.com (P.Z.); renxiaopeng2022@163.com (X.R.); 18233283771@163.com (S.Z.); alimengyuuuu@163.com (M.L.); 18863095769@163.com (Z.R.); 18632258393@163.com (M.Y.); malinfei2001@163.com (L.M.); liuzi68686@163.com (Z.L.); 18231425975@163.com (K.W.); 2 Agronomy College, National Key Laboratory of Wheat and Maize Crop Science, CIMMYT-China (Henan) Joint Center of Wheat and Maize, Henan Agricultural University, Zhengzhou 450002, China

**Keywords:** wheat, *Fusarium* crown rot, GWAS, QTL, protein kinase

## Abstract

*Fusarium* crown rot (FCR), primarily caused by *Fusarium pseudograminearum*, has emerged as a new threat to wheat production and quality in North China. Genetic enhancement of wheat resistance to FCR remains the most effective approach for disease control. In this study, we phenotyped 435 Chinese wheat cultivars through FCR inoculation at the seedling stage in a greenhouse. Our findings revealed that only approximately 10.8% of the wheat germplasms displayed moderate or high resistance to FCR. A genome-wide association study (GWAS) using high-density 660K SNP led to the discovery of a novel quantitative trait locus on the long arm of chromosome 3B, designated as *Qfcr.hebau-3BL*. A total of 12 significantly associated SNPs were closely clustered within a 1.05 Mb physical interval. SNP-based molecular markers were developed to facilitate the practical application of *Qfcr.hebau-3BL*. Among the five candidate FCR resistance genes within the *Qfcr.hebau-3BL*, we focused on *TraesCS3B02G307700,* which encodes a protein kinase, due to its expression pattern. Functional validation revealed two transcripts, *TaSTK1.1* and *TaSTK1.2*, with opposing roles in plant resistance to fungal disease. These findings provide insights into the genetic basis of FCR resistance in wheat and offer valuable resources for breeding resistant varieties.

## 1. Introduction

*Fusarium* crown rot (FCR) is caused by infections from multiple *Fusarium* pathogens, including *F. pseudograminearum*, *F. graminearum*, *F. culmorum*, and *F. avenaceum*. In the Huanghuai wheat-growing region of North China, *F. pseudograminearum* has been identified as the predominant species [1]. *Fusarium* pathogens have a wide range of hosts, including wheat, barley, maize, and others. They can persist in returned straw debris and soil for up to five years [2]. The extensive and long-term implementation of wheat–maize rotation and straw incorporation practices in North China has significantly altered the bio-chemical characteristics of the soil [3], as well as the disease-suppressing capacity of the soil microbiome [4]. Consequently, FCR has emerged as an increasing threat to wheat production in these areas.

Rationally utilizing wheat germplasms that exhibit resistance to FCR and exploring the genetic basis underlying this resistance are essential strategies for effectively combating this fungal disease. These approaches can be complemented by implementing sound crop rotation practices. It is worth noting that certain FCR pathogens, such as *F. graminearum*, can also induce another severe fungal disease in wheat spikes, known as *Fusarium* head blight (FHB). There appears to be no correlation between wheat resistance to FCR and FHB, neither in terms of resistant germplasms nor genetic loci [5,6]. However, an identified FHB-resistance gene, *Fhb7*, which encodes a glutathione S-transferase (GST), has demonstrated broad-spectrum resistance to FCR caused by *F. pseudograminearum* through the detoxification of trichothecenes [7]. Over the past decade, there has been extensive research on the genetic loci controlling FCR resistance, with some loci being closely clustered on wheat chromosomes 2DS, 3BL, 4BS, 4BL, and 5DS [8]. Recently, researchers have utilized an EMS mutant of the wheat variety “Chuannong 16” to construct a genetic segregating population for the identification of a novel FCR resistance locus, designated as *Qfcr.cau-2A* [9]. Using 358 wheat germplasm resources and 55K SNP chip data, researchers identified quantitative trait locus (QTL) on the 5D chromosome [10]. In another study, utilizing 209 Chinese wheat germplasm resources and 55K SNP chip data, multiple major FCR resistance QTLs were identified on chromosomes 2A, 5A, and 7D in common wheat [11]. In a recent study, researchers rapidly assessed FCR resistance in 223 Chinese wheat germplasm resources using the inoculation method of infiltration into the base of seedling stems. By combining this with existing 90K SNP chip data, a novel disease-resistant genetic locus, *Qfcr.cau.3D-3*, was identified [12].

In particular, several quantitative trait loci (QTLs) controlling resistance to FCR have been mapped to wheat chromosome 3B. Notably, a significant QTL for FCR resistance was identified in the distal region of chromosome 3BS, close to the FHB resistance locus *Fhb1* [13]. *Fhb1* has been successfully cloned and is known to encode a putative histidine-rich calcium-binding protein [14]. A genome-wide association study (GWAS) was conducted on a set of 126 advanced lines of spring bread wheat obtained from the International Maize and Wheat Improvement Center (CIMMYT). The study identified two marker-trait associations (MTAs) located on chromosome 3B, which are associated with the FCR phenotype observed in the field [15]. Another GWAS on 475 wheat genotypes using a 9K SNP array identified three MTAs on chromosome 3BL [16]. The Australian spring wheat cultivar ‘Sunco’, which exhibits partial resistance to FCR, has been extensively studied. Multiple QTLs, including two major ones, *Qcr.usq-3B.1* and *Qcrs.wsu-3BL*, were closely detected in the distal region of chromosome 3BL [5,17,18,19]. Furthermore, two SSR markers on chromosome 3BS were found to be associated with FCR resistance in ‘Sunco’ [18]. A series of studies were conducted on a *Triticum spelta*-derived genotype, called ‘CSCR6′, which exhibited relatively high resistance to FCR. A major QTL, *Qcrs.cpi-3B*, responsible for FCR resistance in ‘CSCR6′, was mapped to the distal end of chromosome 3BL [20,21].

In this study, we aimed to identify genetic factors associated with FCR resistance and predict candidate-resistant genes to gain insights into the genetic improvement of wheat resistance to FCR. This study should be able to provide valuable insights into the genetic basis of wheat resistance to FCR and offer potential targets for further genetic improvement of wheat cultivars with enhanced resistance to this destructive disease.

## 2. Results

### 2.1. Evaluation of FCR Resistance in 435 Chinese Wheat Germplasms Seedlings

**Figure 1:**
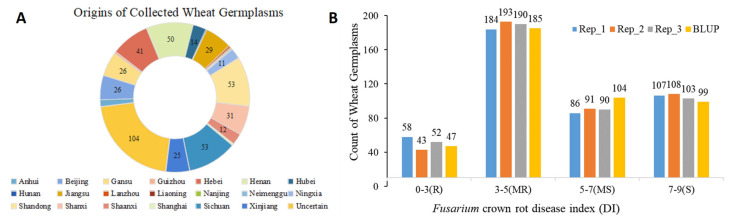
Evaluation of FCR resistance in collected Chinese wheat germplasms. (**A**) Geographical origin or major planting area of the collected Chinese wheat germplasms. (**B**) Distribution of FCR resistance levels among the collected Chinese wheat germplasms. R, resistance; MR, moderate resistance; MS, moderate susceptible; S, susceptible.

Seedlings of 435 Chinese wheat germplasms collected from at least 20 provinces across China were cultivated in a greenhouse (Figure 1A) and inoculated with the dominant strain of *F. pseudograminearum* isolated from Hebei Province, China. The phenotypes were examined at 28 days post-inoculation (dpi). BLUP values for the disease index (DI) of each wheat germplasm were calculated based on three independent assays (Table S1). The correlation coefficients between the three independent assays and BLUP values ranged from 0.94 to 0.97 (Table S2). Out of the 435 Chinese wheat lines evaluated, only 47 genotypes (10.8%) displayed a DI lower than 3.0. Among these, five accessions (hk1/6/nvsr3/5bez/tvr, Lan 14, Long Jian 127, Lu Mai 21, and Ning Mai 15 Hao) exhibited an average DI lower than 1.0 (Table 1 and Figure 1B). The remaining 388 genotypes displayed DIs higher than 3.0, with 203 genotypes (46.7%) having DIs higher than 5.0 (Figure 1B), indicating that most genotypes were highly susceptible to FCR disease.

### 2.2. A Novel FCR-Resistant QTL on the Long Arm of Chromosome 3B Was Identified through GWAS

**Figure 2:**
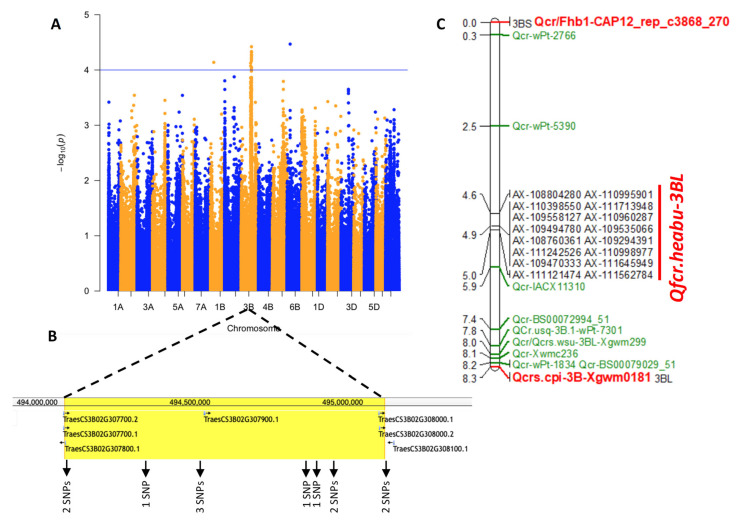
GWAS analysis reveals a novel FCR-resistant QTL on chromosome 3BL. (**A**) Manhattan plot showing associations between SNPs and FCR resistance identified by GWAS using the mix linearized model (MLM). (**B**) Physical interval of approximately 1.05 Mb on chromosome 3BL containing twelve associated SNPs. (**C**), Distribution map showing associated SNPs and previously reported FCR-resistant QTLs on chromosome 3B. QTLs with great effect were colored in red, others were in green.

Genotyping was conducted using the wheat 660K SNP array [22]. After filtering, a total of 399,739 SNPs distributed across all 21 chromosomes of wheat were utilized for GWAS. Among them, 18 SNPs exhibited significant associations (*p* value < 0.0001) with FCR resistance by GWAS using the mix linearized model (MLM) (Table 2, Figure 2 and Figure S1), with 16 of these SNPs being located on the long arm of chromosome 3B (Figure 2A). In the Chinese Spring reference genome (IWGSC RefSeq v1.1), 12 of these SNPs were found to reside within a physical interval of approximately 1.05 Mb on chromosome 3BL (494,074,512 bp to 495,123,078 bp), which was designated as *Qfcr.hebau-3BL* (Figure 2B). The physical distributions of all associated SNPs were estimated and compared with previously reported FCR resistance QTLs on chromosome 3B (Figure 2C), revealing that *Qfcr.hebau-3BL* represents a novel locus positioned near the centromere region of chromosome 3BL.

### 2.3. Design of CAPS/dCAPS Markers Associated with Qfcr.hebau-3BL

**Figure 3:**
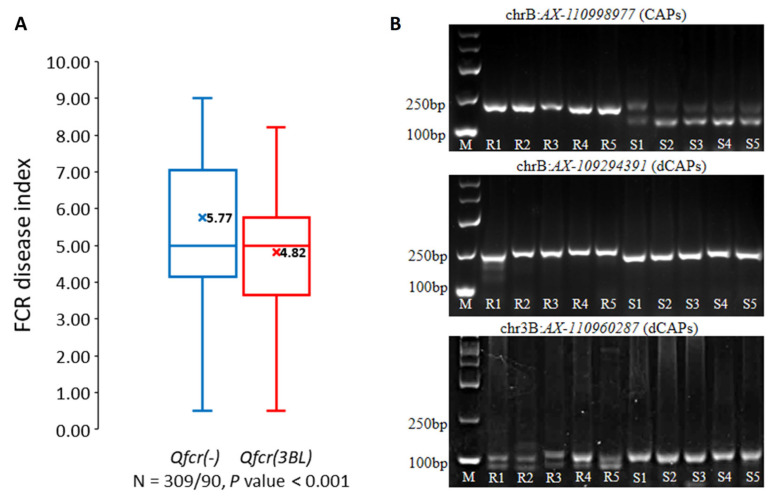
Phenotypic effects of *Qfcr.hebau-3BL* and development of CASP/dCAPS markers. (**A**) Phenotypic effects of wheat germplasms with or without *Qfcr.hebau-3BL* on FCR resistance. The key SNP *AX-110960287* within the *Qfcr.hebau-3BL* interval was used to genotype the collected germplasms. Accessions with the *Qfcr(3BL)* haplotype exhibited significantly lower average DI values compared to accessions with the *Qfcr(-)* haplotype (*p* < 0.001). The horizontal line in the distribution chart represents the median of the data distribution, while the cross-shaped marker indicates the mean value of the data. (**B**) Development and validation of SNP-based CAPS/dCAPS markers for *Qfcr.hebau-3BL*. M: DNA marker DL2000, R1–R5: resistant lines with *Qfcr(3BL)* haplotype, S1–S5: susceptible lines with *Qfcr(-)* haplotype.

Haplotype analysis was performed for the 12 associated SNPs within the *Qfcr.hebau-3BL* interval on chromosome 3BL. Based on the genotypes of resistant and susceptible wheat germplasms, two main haplotypes, *Qfcr(-)* and *Qfcr(3BL)*, were identified from these 12 associated SNPs (Table S3). By referring to the genotype of a key SNP *AX-110960287* within the *Qfcr.hebau-3BL* interval, it was found that 90 accessions with the *Qfcr(3BL)* haplotype exhibited a significantly (*p* < 0.001) lower average DI value (4.82) compared to the other 309 accessions with the *Qfcr(-)* haplotype (5.77) (Figure 3A). This difference in the disease index suggests the potential association between the *Qfcr(3BL)* haplotype and increased FCR resistance.

To validate the findings of GWAS, CAPS/dCAPS markers associated with *Qfcr.hebau-3BL* were designed based on the associated SNPs (Table S4). One CAPS and two dCAPS markers were successfully developed to detect the SNP allele in wheat genotypes with or without *Qfcr.hebau-3BL* (Figure 3B). These molecular markers provide a reliable tool for identifying and characterizing the *Qfcr.hebau-3BL* locus in different wheat genotypes.

### 2.4. Prediction of Candidate FCR-Resistant Genes within Qfcr.hebau-3BL

Based on the association analysis, annotated genes located within the 1.05 Mb *Qfcr.hebau-3BL* interval were identified as candidate FCR-resistant genes. Among these genes, five genes were of particular interest based on gene annotations in the Chinese Spring reference genome, with two of them exhibiting alternative splicing (Figure 2B). These genes include *TraesCS3B02G307700*, which is predicted to encode a serine/threonine protein kinase A-Raf-like protein (designated as *TaSTK1*). Additionally, *TraesCS3B02G308100* and *TraesCS3B02G307900* are predicted to encode a chaperone ClpB1-like protein and mannan endo-1,4-mannosidase 2-like protein, respectively. *TraesCS3B02G307800* and *TraesCS3B02G308000* are predicted to encode uncharacterized proteins.

**Figure 4:**
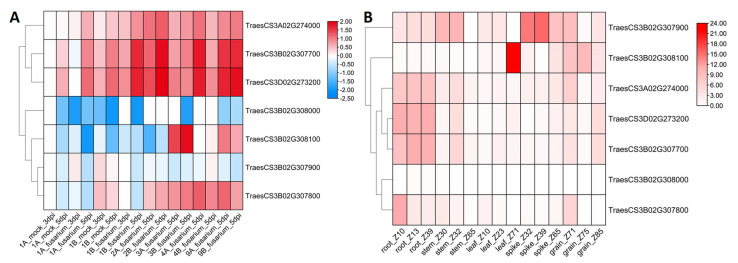
Prediction of candidate resistance genes within the *Qfcr.hebau-3BL* interval. (**A**) Expression patterns of five annotated genes within the *Qfcr.hebau-3BL* interval and homologs of *TaSTK1* in A and D sub-genomes during wheat resistance response to FCR infection. (**B**) Expression patterns of candidate genes in different tissues and developmental stages.

To further investigate the expression levels of these candidate genes, we examined their expression patterns using published RNA-seq data on wheat-resistant responses to FCR [23]. Compared to the mock treatment with double-distilled water, the expression levels of *TraesCS3B02G307700* (*TaSTK1*) and its homologs in the A and D sub-genomes were significantly induced during FCR infection (Figure 4A). Furthermore, according to the expression data of wheat development [24], transcripts of *TaSTK1* and its homologs were found to be highly accumulated in root and stem tissues (Figure 4B). Considering these expression patterns and gene annotations, *TaSTK1* was selected for further investigation of its function in FCR resistance.

**Figure 5:**
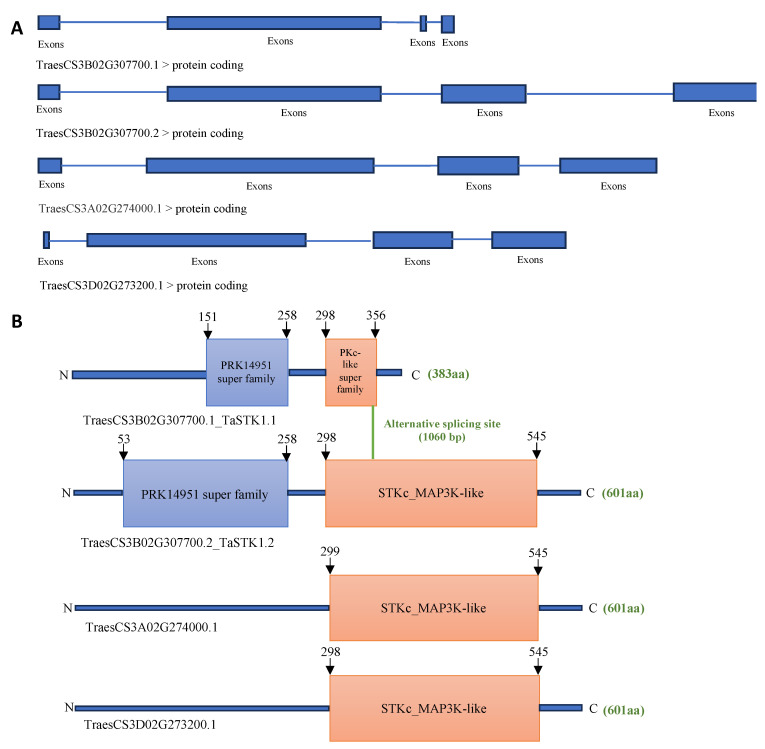
Alternative splicing transcripts of *TaSTK1.* (**A**) Schematic representation of the two alternative splicing transcripts of *TaSTK1* and its homologs in A and D sub-genomes. (**B**) Predicted domain structures of the proteins encoded by the two transcripts of *TaSTK1* and its homologs in A and D sub-genomes.

This gene exhibits two alternative splicing transcripts exclusively in the B genome copy, named as *TaSTK1.1* and *TaSTK1.2*, encoding two different versions of protein kinase with lengths of 383 amino acids (aa) and 601 aa, respectively (Figure 5A,B). Phylogenetic analysis revealed that the TaSTK1 protein is conserved among different plant species, with its closest homologs in *Arabidopsis* and rice being RAF36 (AT5G58950.1) and HT1 (XP_015622210.1), respectively (Figure S2).

### 2.5. Opposing Functions of TaSTK1.1 and TaSTK1.2 against Fungal Disease

**Figure 6:**
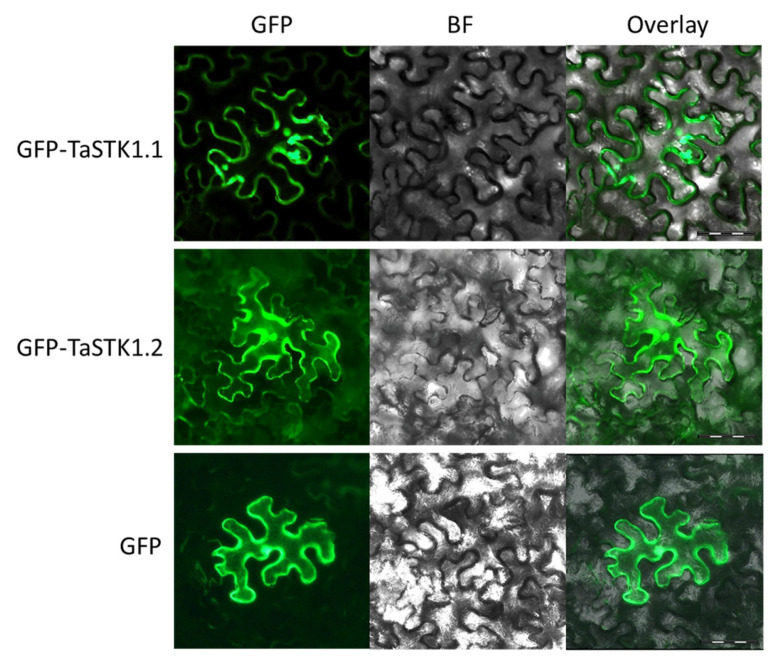
Subcellular localization of GFP-TaSTK1.1 and GFP-TaSTK1.2 transiently expressed in *Nicotiana benthamiana*, visualized by confocal microscopy at 3 dpi.

To gain initial insights into the functions of TaSTK1.1 and TaSTK1.2, the N-terminus of these proteins was fused with green fluorescent protein (GFP) driven by the *35S* promoter. *GFP-TaSTK1.1* and *GFP-TaSTK1.2* constructs were transiently expressed in the leaves of *N. benthamiana* and visualized using confocal microscopy. Like the control of free GFP, the GFP signals of both GFP-TaSTK1.1 and GFP-TaSTK1.2 were observed throughout the cell, including the nucleus and cytoplasm in *N. benthamiana* (Figure 6). This suggests that the different alternative splicing versions of *TaSTK1* do not appear to influence the subcellular localization of the encoded proteins.

**Figure 7:**
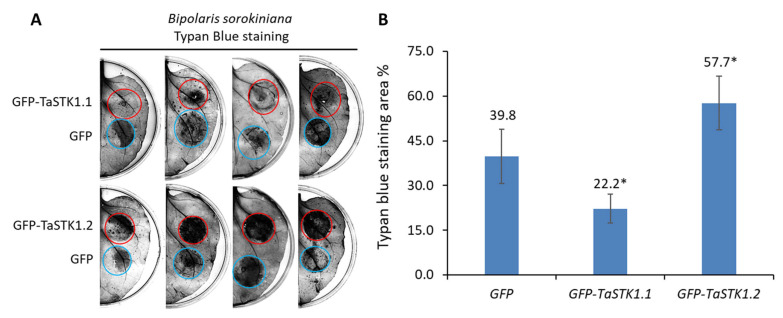
Functional analysis of *TaSTK1.1* and *TaSTK1.2* in plant resistance to fungal infection. (**A**) Trypan blue staining of *N. benthamiana* leaves expressing GFP-TaSTK1.1 and GFP-TaSTK1.2, followed by challenge with *Bipolaris sorokiniana* at 2 dpi. (**B**) Statistical analysis of the proportion of the trypan blue staining region on *N. benthamiana* leaves infected with *B. sorokiniana*. Leaves expressing free GFP served as the negative control. The asterisk indicated a significant difference (*p* < 0.05) between the GFP-fused TaSTK1 and the free GFP control.

Since we did not observe any successful infection of *F. pseudograminearum* on tobacco leaves (Figure S3), to investigate the roles of *TaSTK1* in plant resistance to necrotrophic fungal pathogens, *GFP-TaSTK1.1* and *GFP-TaSTK1.2* were transiently expressed in *N. benthamiana* leaves, followed by inoculation with *Bipolaris sorokiniana* at 2 days post-infiltration. Plant resistance was evaluated by measuring the proportion of the trypan blue staining area in the filtrated leaf region. Compared to leaves infiltrated with free GFP, the expression of GFP-TaSTK1.2 significantly (*p* = 0.023, N = 10) enhanced the virulence of *B. sorokiniana* in *N. benthamiana*. Conversely, the expression of GFP-TaSTK1.1 significantly (*p* = 0.019, N = 10) attenuated the virulence of *B. sorokiniana* (Figure 7A,B). These results indicate that *TaSTK1.2* renders *N. benthamiana* more susceptible to *B. sorokiniana*, while *TaSTK1.1* promotes plant resistance against *B. sorokiniana*.

## 3. Discussion

*Fusarium* crown rot is a chronic soil-borne disease that causes significant yield loss in wheat [25]. Despite recent attention, limited progress has been made in screening wheat cultivars for FCR resistance. Only a few wheat cultivars have shown high resistance to FCR disease [26], and there is still a need to screen and identify more resistance germplasms and loci. Our study revealed that approximately 10.8% of the evaluated wheat germplasms exhibited moderate or high resistance to FCR disease. Among these, ‘Lan 14’, ‘Long Jian 127’, ‘Lu Mai 21’, and ‘Ning Mai 15 Hao’ had DI values lower than 1.00, demonstrating great potential for FCR resistance breeding. Hou et al. [26] identified 27 highly resistant landraces from 361 Chinese wheat landraces for FCR resistance breeding. Although a minority of wheat cultivars showed moderate or high resistance to FCR, these findings represent significant breakthroughs in improving FCR resistance. Our study enriches the pool of resistant resources for FCR disease and highlights a few highly resistant cultivars.

Our previous study has summarized putative genetic loci associated with wheat resistance to FCR disease [8]. These loci are distributed across various chromosomes, including several QTLs identified on chromosome 3B. For instance, *Qcr-IACX11310* was located on the long arm of chromosome 3B, at a considerable distance from *Qfcr.hebau.3BL*. Hence, *Qfcr.hebau.3BL* represents a novel QTL for wheat resistance to FCR disease. Other studies have also identified additional QTLs associated with FCR resistance. Among the reported QTLs, *Qcr/Fhb1-3BS-CAP12_rep_c3868_270* and *Qcrs.cpi-3B-Xgwm0181* are located on 3BS and 3BL, respectively. These stable QTLs have large effects or are linked to specific genes [13,20,27]. The near-isogenic lines carrying *Qcrs.cpi-3B-Xgwm0181* have been developed for breeding programs [20]. The mapping of *Qcrs.cpi-3B* identified 63 coding genes in the reference wheat genome [21]. Subsequent cloning and functional validation of candidate genes will help elucidate the molecular mechanisms underlying wheat resistance to FCR disease.

In our study, based on RNA-seq data from infectious samples, five high-confidence genes were identified within the *Qfcr.hebau-3BL* interval. Among them, *TraesCS3B02G307700*, which encodes a protein kinase named *TaSTK1*, was significantly upregulated during the infectious stages at 5 dpi. In *Arabidopsis*, group C Raf-like protein kinases were found to negatively regulate abscisic acid (ABA) signaling and serve as direct substrates of SnRK2s, central regulators in ABA signaling and stress responses in plants [28]. Raf-like proteins, a subfamily of mitogen-associated protein kinase kinase kinase (MAPKKKs), also play significant roles in plant–pathogen interactions [29]. For instance, in *Arabidopsis*, the Raf-like kinase EDR1 negatively regulates plant resistance against bacteria, fungi, and oomycetes [30]. Similarly, the rice Raf-like kinase ILA1 suppresses the MAPKK4-MPK6 cascade-mediated resistance against bacterial blight [31]. However, the functions of Raf-like kinases in wheat resistance to plant pathogens remain unclear, and further evidence is needed to elucidate the molecular mechanisms underlying plant resistance against pathogens.

Alternative splicing, a process by which a single gene produces multiple protein isoforms, can enhance protein diversity. The *TaSTK1* gene exhibits two transcripts, *TaSTK1.1* and *TaSTK1.2*, which play opposing roles in plant resistance to a fungal pathogen. Similar cases have been reported, such as the LAMMER kinase gene *OsDR11* in rice, which has long (*OsDR11L*) and short (*OsDR11S*) transcript variants with opposite roles in resistance against *Xanthomonas oryzae* pv. *oryzae* (*Xoo*) [32]. Analysis of jasmonic acid (JA) levels and the relationship between these two transcripts suggested that *OsDR11L* acts as a negative regulator of rice resistance, potentially suppressing JA signaling, while *OsDR11S* may inhibit the function of *OsDR11L*, leading to resistance [32]. In our study, we characterized the preliminary functions of the two alternatively spliced transcripts of *TaSTK1* and found that *TaSTK1.1* promotes plant resistance, while *TaSTK1.2* may act as a negative regulator of plant resistance against *B. sorokiniana*. Further studies are urgently needed to investigate the mechanisms of action of *TaSTK1.1* and *TaSTK1.2* in wheat resistance to FCR disease.

## 4. Materials and Methods

### 4.1. Wheat Planting and FCR Inoculation

A total of 435 Chinese wheat germplasms were evaluated for FCR resistance (Table S1). All the seeds were harvested from the fields of Hebei Agricultural University (N151.424779, E38.814959) during the years 2020–2021. The seeds were then grown in a greenhouse under controlled conditions of 25 °C temperature and a 13 h light/11 h dark cycle. The dominant strain of *F. pseudograminearum* in Hebei Province, China, was isolated from wheat plants infected in the fields in the year 2020 [33]. The fungal pathogen was first cultured on potato dextrose agar (PDA) plates at a temperature of 25 °C for a duration of 5 days. Subsequently, the *F. pseudograminearum* was inoculated in the wheat grain media and cultured for an additional 10 days to prepare for the inoculation assay. To inoculate *F. pseudograminearum* on the base of wheat stems, each germplasm was planted with eight seeds mixed with three blocks (approximately 1 cm^3^ each) of the *F. pseudograminearum*-inoculated wheat grain media in plots with a depth of 1–2 cm.

The FCR symptoms of the 435 Chinese wheat germplasms were evaluated in three independent assays conducted in a greenhouse. Disease severity was assessed at 28 days post-inoculation (dpi) to determine the disease index (DI), which was measured on a scale of 0 to 9, where 0 represents no obvious symptoms and 9 represents complete necrotic symptoms, as described previously [34]. Statistical analyses for Best Linear Unbiased Predictor (BLUP) values were performed using Python software v3.12.2.

### 4.2. GWAS Procedure

Genomic DNA was extracted from fresh leaf tissues of each accession using the modified cetyltrimethylammonium bromide (CTAB) method [35]. All 435 accessions were genotyped with a high-density wheat SNP array (660 K) conducted by Beijing CapitalBio Corporation [22]. The SNP dataset was filtered based on a call rate > 0.85 and a minor allele frequency (MAF) < 0.05. Population structure analysis (Q matrix) was performed using STRUCTURE software v2.3.4 with unlinked markers (*r*^2^ = 0). The number of subpopulations (k) was determined using five independent runs with a burn-in of 1000 iterations followed by 1000 Monte Carlo Markov Chain (MCMC) replicates, considering a range of 1–10 as putative values [36]. The optimal alignment of the five replicates was obtained using CLUMPP [37]. The population structure analysis revealed that the 435 wheat materials were divided into three subgroups. The first subgroup predominantly consisted of materials from provinces such as Henan, Anhui, Jiangsu, and Shanxi. The second subgroup mainly comprised farmer varieties. The third subgroup was primarily composed of materials from Beijing, Gansu, Shandong, and overseas sources.

Kinship analysis was conducted using the Genome Association and Prediction Integrated Tool (GAPIT) package v2 [38]. The Principal Component Analysis (PCA) results indicate that all varieties can also be divided into three major subgroups, with a clear distinction between farmer varieties and the rest of the cultivated varieties. GWAS was conducted using the mixed linear model (PCA + K) in R software v4.3.2 with GAPIT, and the kinship matrix (K) was calculated using the VanRaden method [38,39]. The linkage disequilibrium (LD) parameter (*r*^2^) between pairwise SNPs (MAF > 0.05) was estimated using TASSEL 3.0 software. The observed *p*-values were plotted against the expected *p*-values (Figure S4). The threshold for the *p* value was determined using a modified Bonferroni correction (Genetic type 1 Error Calculator, version 0.2), with a suggestive threshold of *p* value = 1.0 × 10^−4^ (*p* = 1/n, n = effective SNP number). A total of 18 SNPs were obtained from the 435 Chinese wheat cultivars, and these SNPs were mapped to the Chinese Spring physical reference map IWGSC RefSeq v1.1.

### 4.3. Design and Validation of CAPS/dCAPS Markers

Based on the SNP information obtained from GWAS, molecular markers using the CAPS/dCAPS technique were developed. For the CAPS marker, the DNA sequence of interest was analyzed to identify potential restriction enzyme recognition sites. PCR primers are designed to amplify the specific region containing the potential CAPS sites. The primers are positioned to flank the target site to ensure amplification of the desired fragment. For the dCAPS marker, a mismatch was introduced to the primer near the SNP to create a distinguished recognition of the restriction enzyme. The reliability of these molecular markers was validated by analyzing the correlation between the detected genotypic signals and sequenced SNP data. Genomic DNA was extracted from five resistant lines with *Qfcr.hebau-3BL* and five susceptible lines without *Qfcr.hebau-3BL.* PCR products were digested by the corresponding restriction enzyme and then separated using gel electrophoresis.

### 4.4. Prediction of Candidate FCR-Resistant Genes

High-confidence annotated genes, located within the physical interval that clustered with the associated SNPs on chromosome 3BL, were identified using the WheatOmics website [40]. Expressions of candidate FCR resistance genes and their homologs in various transcriptomes were gathered from the same website. Additional homologs of TaSTK1 were collected from the GenBank database on the NCBI website (https://www.ncbi.nlm.nih.gov, accessed on 15 March 2023). Alignment of the TaSTK1 homologs was performed using the MUSCLE method in MEGA software v7.0.14, and a neighbor-joining tree was generated to analyze the relationships between the sequences.

### 4.5. Transient Expression of GFP-TaSTK1.1 and GFP-TaSTK1.2

Two transcripts of *TraesCS3B02G307700*, namely *TaSTK1.1* and *TaSTK1.2*, were initially isolated from the cDNA of the common wheat cultivar ‘Lu Mai 21’, which carries the *Qfcr.hebau-3BL* locus (primers in Table S5). These transcripts were subsequently cloned into the pBIN vector (*35S::GFP-gene*) to generate *GFP-TaSTK1.1* and *GFP-TaSTK1.2* constructs, respectively. The sequenced vectors were then transformed into *Agrobacterium tumefaciens* strain GV3101 and transiently expressed in *Nicotiana benthamiana* plants. After agroinfiltration, fluorescence was observed 2 days later using a confocal microscope (Nikon A1, HD25 confocal microscope, Tokyo, Japan).

*GFP-TaSTK1.1* and *GFP-TaSTK1.2* constructs were agroinfiltrated into *Nicotiana benthamiana* leaves. The leaves were detached at 2 dpi and placed in a plastic tray covered with moist filter paper, with the petioles wrapped in wet cotton for hydration. For the inoculation assay, the *N. benthamiana* leaves were inoculated with a 5 cm diameter fungal disc of the dominant strain of *Bipolaris sorokiniana* isolated in Hebei Province, China. The inoculated leaves were then incubated in a growth chamber at a temperature of 25 °C for a duration of 5 days. For trypan blue staining assays, the inoculated *N. benthamiana* leaves were stained using a trypan blue solution composed of 10 g phenol, 10 mL glycerol, 10 mL lactic acid, 10 mL water, and 10 mg of trypan blue. The samples were boiled for two minutes, followed by cooling to room temperature and distaining with a 2.5 g/mL chloral hydrate solution. After rinsing with water, the samples were photographed. The resistance of *N. benthamiana* to *B. sorokiniana* was assessed by quantifying the proportion of the infiltrated leaf region stained with trypan blue. The mean and standard error were calculated using Microsoft Excel software v2021. The Dunnett test was performed using SAS software v9.4.

## 5. Conclusions

In conclusion, our study evaluated FCR resistance in a large collection of Chinese wheat cultivars and found that only around 10.8% of them displayed moderate or high resistance to FCR. Through GWAS analysis, we identified a novel QTL, *Qfcr.hebau-3BL*, on chromosome 3BL associated with FCR resistance. Three CAPS/dCAPS markers were developed based on closely clustered SNPs within this QTL for efficient breeding applications. The corresponding genomic region contained five annotated genes, among which *TraesCS3B02G307700,* encoding a protein kinase, was selected for further validation. Alternative splicing of *TraesCS3B02G307700* produced two transcripts, *TaSTK1.1* and *TaSTK1.2*, which showed opposing roles in plant resistance against fungal pathogen. This study contributes valuable FCR-resistant germplasms and identifies a genetic locus for improving wheat resistance to FCR.

## Figures and Tables

**Table 1:** Wheat germplasms with moderate or higher resistance to FCR disease.

Resistance Level	Range of Disease Index	Name of Wheat Germplasms
Resistance (R)	DI < 1.0	hk1/6/nvsr3/5bez/tvr, Lan 14, Long Jian 127, Lu Mai 21, Ning Mai 15 Hao
Moderate Resistance (MR)	1.0 < DI < 3.0	Bai He Shang ZM1709, Zheng Mai 366, Xi Ke Mai 2 Hao, nuwest/4/d887-74/pew/, Shan Nong 20, Xu Mai 29, Hong Ban Mang ZM1685, WGRC10/3/KS93U69/sib/TA2455//KS93U69/4/JAGGER, Lu Mai 14, Lu Mai 1 Hao, Shi Dong 7 Hao, MV Laura, Lu Mai 7 Hao, Rong Mai 3 Hao, Lu Mai 12 Hao, Luo Han 2 Hao, Shi Luan 02-1, mv05-08, Bai Mang Bian Sui ZM1852, kanto 107, Tian 95HF2, Zhong Liang 24, A Bo, Luo Pang Tou ZM5962, Tu Mai ZM1950, Huai Mai 21, Xin Mai 13, Lang Yan 43, Lan 13, Yu Lin Bai ZM1686, Bai Nong 64, Nong Da 211, Ma Zha Tou ZM1756, Yu Mai 53, 98039g5-103, Chuan Yu 16, Lan 17, Lai Yang Qiu ZM1747, aca 801, Naxos (x3), Shi You 20, w014204

**Table 2:** SNPs significantly associated with FCR resistance revealed by GWAS.

Marker	Chromosome	Physical Position	*F* Value	*p* Value
AX-110654128	1B	159389204	9.75605	7.28 × 10^−5^
AX-108804280	3B	456983079	9.73002	7.49 × 10^−5^
AX-110995901	3B	457756000	9.59965	8.45 × 10^−5^
AX-110398550	3B	490095055	9.90892	6.36 × 10^−5^
AX-111713948	3B	490587664	9.48509	9.43 × 10^−5^
AX-109558127	3B	494074512	9.97258	5.92 × 10^−5^
AX-110960287	3B	494078054	9.82021	6.85 × 10^−5^
AX-109494780	3B	494193152	17.35716	3.79 × 10^−5^
AX-109535066	3B	494528567	9.54042	8.94 × 10^−5^
AX-108760361	3B	494529145	10.22797	4.64 × 10^−5^
AX-109294391	3B	494529808	10.09041	5.29 × 10^−5^
AX-111242526	3B	494705774	9.43673	9.91 × 10^−5^
AX-110998977	3B	494812675	10.05171	5.49 × 10^−5^
AX-109470333	3B	494966841	10.21113	4.73 × 10^−5^
AX-111645949	3B	494984964	10.09931	5.25 × 10^−5^
AX-111121474	3B	495105281	9.92331	6.23 × 10^−5^
AX-111562784	3B	495123078	10.20079	4.77 × 10^−5^
AX-111283740	6B	151370087	10.55803	3.40 × 10^−5^

## Data Availability

All data, models, or code generated or used during the study are available from the corresponding author on request.

## Supplementary Materials

The following supporting information can be downloaded at: https://www.mdpi.com/article/10.3390/plants13060856/s1; Table S1. FCR phenotype of tested Chinese common wheat germplasms; Table S2. Pearson correlations of Fusarium crown rot reactions in three trials and BLUP; Table S3. Haplotype analysis on 12 SNPs within the *Qfcr.hebau-3BL* physical interval; Table S4. CAPS/dCAPS markers were designed based on the SNPs associated with *Qfcr.hebau-3BL*; Table S5. Primers used in this study; Figure S1. Distribution map showing associated SNPs and previously reported FCR resistant QTLs on chromosome 1B and 6B; Figure S2. Phylogenetic analysis of TaSTK1 and other serine/threonine protein kinase A-Raf-like proteins. Figure S3. Inoculation of *Fusarium pseudograminearum* on tobacco leaves. Figure S4. Q-Q plots for GWAS analysis.
