# Supplementary material for: Genome-Wide Association Studies on Chinese Wheat Cultivars Reveal a Novel Fusarium Crown Rot Resistance Quantitative Trait Locus on Chromosome 3BL
Source: Plants (Basel). 2024 Mar 15;13(6):856. doi: 10.3390/plants13060856 (PMC10974656; doi:10.3390/plants13060856)
Supplement: Supplementary file 1 [file plants-13-00856-s001.zip › Suppl Figs.pptx]

## Slide 1
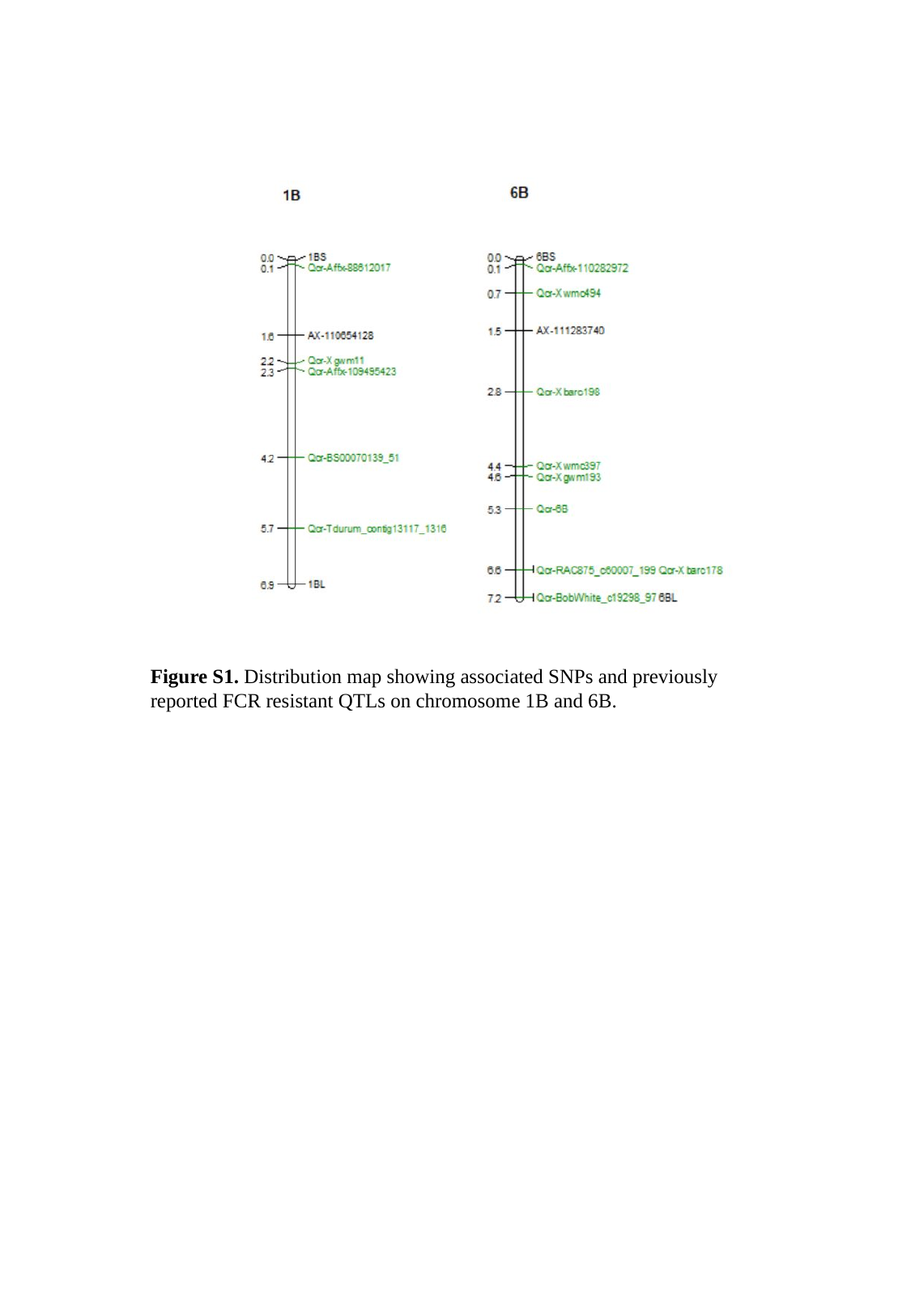

Figure S1. Distribution map showing associated SNPs and previously reported FCR resistant QTLs on chromosome 1B and 6B.

## Slide 2
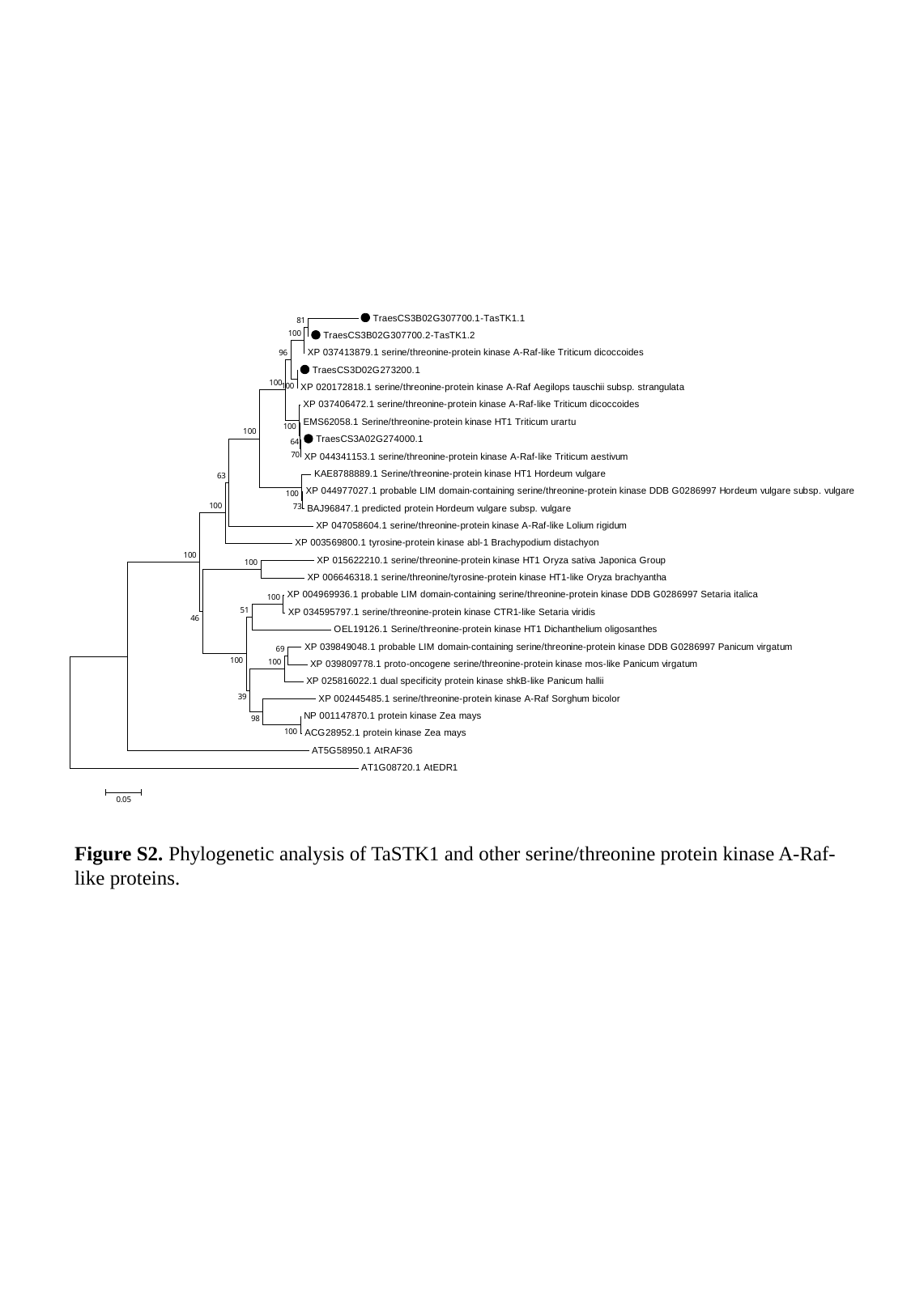

Figure S2. Phylogenetic analysis of TaSTK1 and other serine/threonine protein kinase A-Raf-like proteins.

## Slide 3
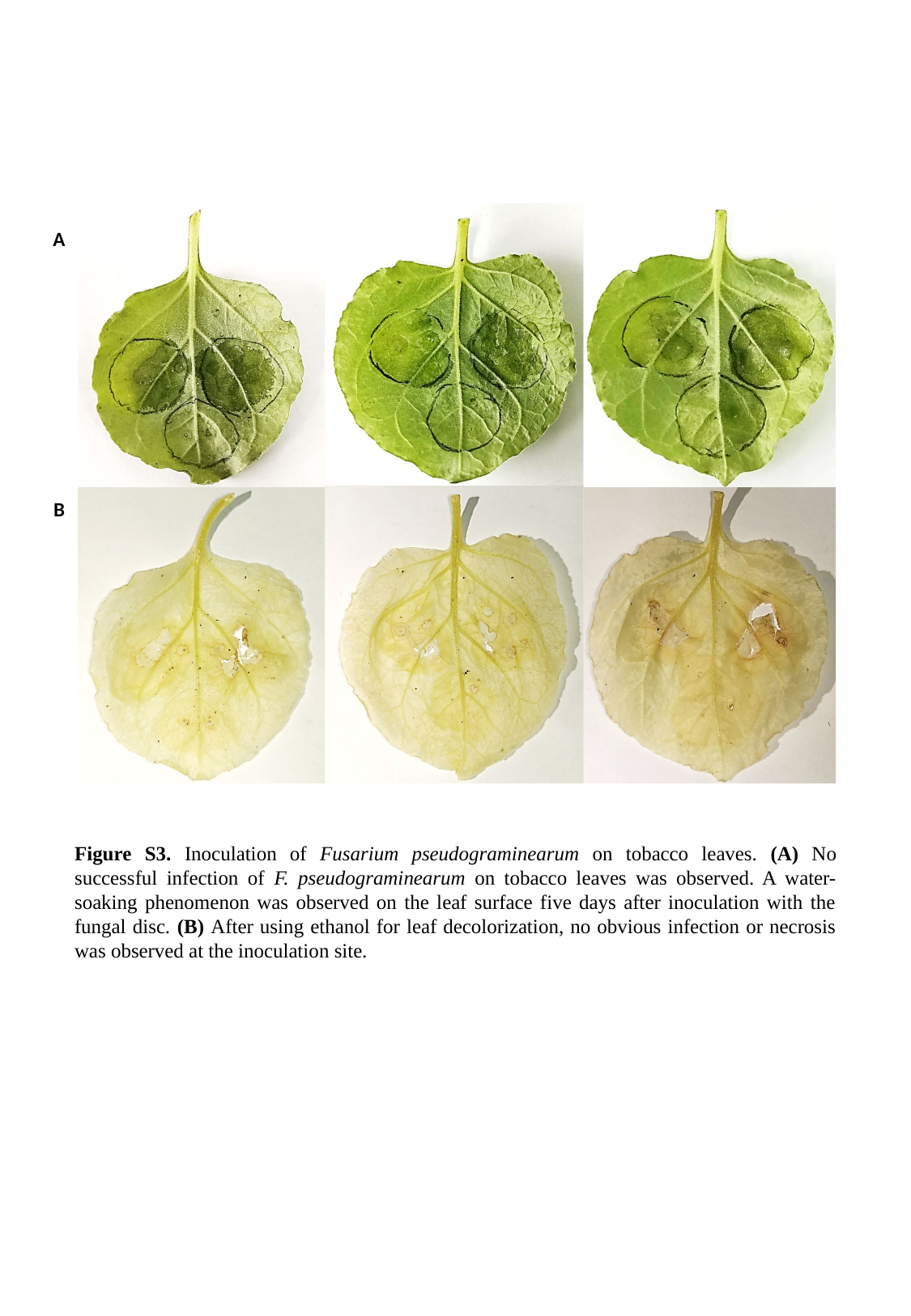

A
B
Figure S3. Inoculation of Fusarium pseudograminearum on tobacco leaves. (A) No successful infection of F. pseudograminearum on tobacco leaves was observed. A water-soaking phenomenon was observed on the leaf surface five days after inoculation with the fungal disc. (B) After using ethanol for leaf decolorization, no obvious infection or necrosis was observed at the inoculation site.

## Slide 4
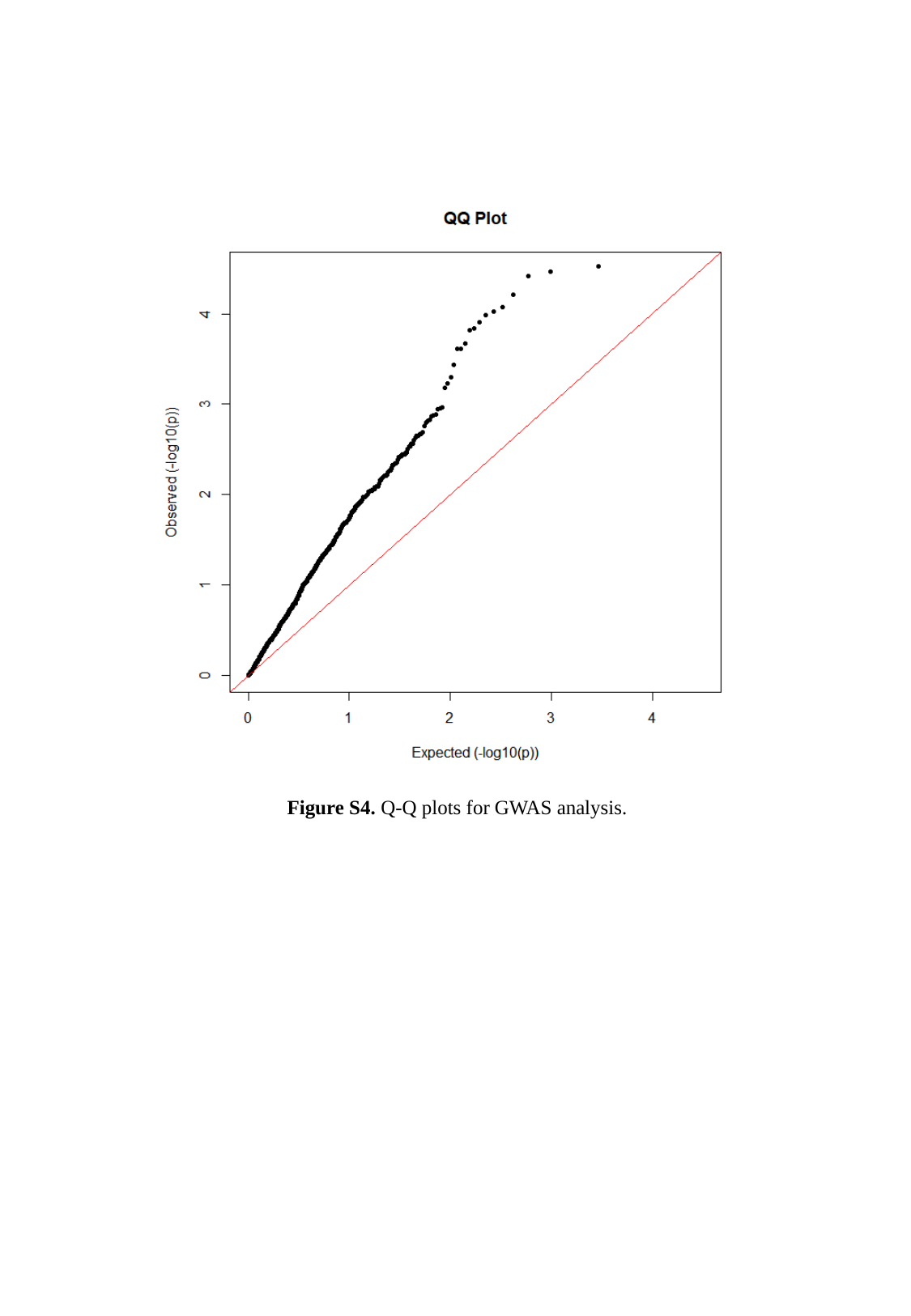

Figure S4. Q-Q plots for GWAS analysis.
