# Supplementary material for: Genome-Wide Association Studies on Chinese Wheat Cultivars Reveal a Novel Fusarium Crown Rot Resistance Quantitative Trait Locus on Chromosome 3BL
Source: Plants (Basel). 2024 Mar 15;13(6):856. doi: 10.3390/plants13060856 (PMC10974656; doi:10.3390/plants13060856)
Supplement: Supplementary file 1 [file plants-13-00856-s001.zip › Suppl Table.pdf]

Table S1. FCR phenotype of tested Chinese common wheat germplasms.

Germplasm No.	Germplasm Name (Pinyin)	Germplasm Name (Chinese)	FCR Phenotype (BLUP, Scale 1-9)
L220	hk1/6/nvsr3/5bez/tvr	hk1/6/nvsr3/5bez/tvr	0.713
L66	Lan 14	兰 14	0.750
L311	Long Jian 127	陇鉴 127	0.813
L289	Lu Mai 21	鲁麦 21	0.940
L423	Ning Mai 15 Hao	宁麦 15 号	1.000
L250	Bai He Shang ZM1709	白和尚 ZM1709	1.200
L193	Zheng Mai 366	郑麦 366	1.450
L141	Xi Ke Mai 2 Hao	西科麦 2 号	1.500
L264	nuwest/4/d887-74/pew/	nuwest/4/d887-74/pew/	1.500
L76	Shan Nong 20	山农 20	1.628
L421	Xu Mai 29	徐麦 29	1.668
L268	Hong Ban Mang ZM1685	红半芒 ZM1685	1.833
L248	WGRC10/3/KS93U69/sib/TA2455//KS93U69/4/JAGGER	WGRC10/3/KS93U69/sib/TA2455//KS93U69/4/JAGGER	1.838
L372	Lu Mai 14	鲁麦 14	1.858
L376	Lu Mai 1 Hao	鲁麦 1 号	1.860
L167	Shi Dong 7 Hao	石冬 7 号	1.868
L262	MV Laura	MV laura	1.898
L111	Lu Mai 7 Hao	鲁麦 7 号	2.000
L112	Rong Mai 3 Hao	蓉麦 3 号	2.000
L114	Lu Mai 12 Hao	鲁麦 12 号	2.000
L124	Luo Han 2 Hao	洛旱 2 号	2.000
L171	Shi Luan 02-1	师栾 02-1	2.000
L228	mv05-08	mv05-08	2.000
L258	Bai Mang Bian Sui ZM1852	白芒扁穗 ZM1852	2.000
L265	kanto 107	kanto 107	2.000
L294	Tian 95HF2	天 95HF2	2.000
L71	Zhong Liang 24	中梁 24	2.000
L461	A Bo	阿勃	2.128
L462	Luo Pang Tou ZM5962	罗旁头 ZM5962	2.128
L469	Tu Mai ZM1950	突麦 ZM1950	2.143
L123	Huai Mai 21	淮麦 21	2.168
L380	Xin Mai 13	新麦 13	2.170
L1	Lang Yan 43	廊研 43	2.250
L99	Lan 13	兰 13	2.250
L460	Yu Lin Bai ZM1686	鱼鳞白 ZM1686	2.290
L48	Bai Nong 64	百农 64	2.423
L16	Nong Da 211	农大 211	2.438
L266	Ma Zha Tou ZM1756	蚂蚱头 ZM1756	2.500
L413	Yu Mai 53	豫麦 53	2.500
L201	98039g5-103	98039g5-103	2.543

L17	Chuan Yu 16	川育 16	2.558
L47	Lan 17	兰 17	2.613
L260	Lai Yang Qiu ZM1747	莱阳秋 ZM1747	2.643
L259	aca 801	aca 801	2.728
L300	Naxos (x3)	Naxos(x3)	3.000
L362	Shi You 20	石优 20	3.000
L477	w014204	w014204	3.000
L153	Fu Yu 3 Hao	福豫 3 号	3.330
L172	Yang Mai 16	扬麦 16	3.330
L369	Ji Han 2 Hao	济旱 2 号	3.330
L90	Jin Mai 54	晋麦 54	3.330
L98	Zi Mai 1 Hao	资麦 1 号	3.330
L174	Ning Dong 1 Hao	宁冬 1 号	3.500
L204	Yu Mai 3 Hao	豫麦 3 号	3.500
L283	Zhong Liang 29	中梁 29	3.500
L3	Nei Jiang 977671	内江 977671	3.500
L364	Wan Mai 38	皖麦 38	3.500
L466	Bai Sha Mai ZM1823	白沙麦 ZM1823	3.500
L130	Yan Nong 24	烟农 24	3.570
L267	Lao Hong Tu Xiao Mai ZM1797	老红土小麦 ZM1797	3.570
L424	Zhou Mai 28	周麦 28	3.570
L122	Chuan Nong 20	川农 20	3.583
L64	Huai Mai 19	淮麦 19	3.600
L139	Su Nong 6 Hao	宿农 6 号	3.670
L194	Wan Mai 19 (Wan Su 8802)	皖麦 19（皖宿 8802）	3.670
L242	Rong Bing Mai ZM1757	荣炳麦 ZM1757	3.670
L253	Hong Huo Mai ZM1681	红火麦 ZM1681	3.670
L381	Chuan Nong 26	川农 26	3.670
L419	Bao Mai 10 Hao	保麦 10 号	3.670
L443	Yan Nong 23	烟农 23	3.670
L447	Xu Mai 25	徐麦 25	3.670
L463	Zao Shi Tian ZM5970	早十天 ZM5970	3.670
L121	Ji Nan 17	济南 17	3.750
L163	Huai Mai 30	淮麦 30	3.750
L175	Shan You 225	陕优 225	3.750
L256	Xi Shan Bian Sui ZM1846	西山扁穗 ZM1846	3.750
L282	Lian Mai 2 Hao	连麦 2 号	3.750
L286	You Mai 2 Hao	优麦 2 号	3.750
L401	Gao You 9908	藁优 9908	3.750
L407	Shi Mai 21 Hao	石麦 21 号	3.750
L418	Lu Mai 11	鲁麦 11	3.750
L454	aztec	aztec	3.750
L472	Lou Gu Ding ZM1673	蝼蛄腚 ZM1673	3.750

L457	Bai Bian Sui ZM1782	白扁穗 ZM1782	3.800
L464	Yao Cheng Dong Zi Mai ZM1807	咬乘肚子麦 ZM1807	3.800
L195	Han 4589	邯 4589	3.858
L10	L224-3	L224-3	3.860
L143	Xian Mai 99	先麦 99	3.860
L159	Luo 6010	漯 6010	3.860
L417	Shan Han 8675	陕旱 8675	3.860
L433	Xi Ke Mai 4 Hao	西科麦 4 号	3.860
L492	Yang Xiao Mai	洋小麦	3.860
L227	Mo Zi Huang ZM5658	茉子黄 ZM5658	3.880
L100	Bi Mai 16	毕麦 16	4.000
L161	Yu Mai 49 (Wen Mai 6 Hao)	豫麦 49（温麦 6 号）	4.000
L2	19HRWSN-39	19HRWSN-39	4.000
L215	re714	re714	4.000
L247	Hong Tou Mai ZM1790	红头麦 ZM1790	4.000
L249	Ban Mang Mai ZM1718	半芒麦 ZM1718	4.000
L333	Xin Dong 18 Hao	新冬 18 号	4.000
L342	Ke Cheng Mai 1 Hao	科成麦 1 号	4.000
L373	Chuan Nong 19	川农 19	4.000
L378	Mian Yang 11	绵阳 11	4.000
L409	Shi You 17	石优 17	4.000
L42	Dong Dong 6 Hao	鄂恩 6 号	4.000
L438	Jin Mai 21	晋麦 21	4.000
L453	Hong Mang Mai ZM1928	红芒麦 ZM1928	4.000
L476	sw	sw	4.000
L12	Zhou Mai 26	周麦 26	4.140
L162	Ke Nong 1093	科农 1093	4.140
L169	He Nong 5290	河农 5290	4.140
L415	Nong Da 139	农大 139	4.140
L437	Lan 25	兰 25	4.140
L442	Yu Mai 8 Hao (Luo Han Yi Hao)	豫麦 8 号(洛旱一号)	4.140
L271	Zhong Liang 22	中梁 22	4.200
L450	Xi Nong 2000	西农 2000	4.200
L170	Nei Xiang 188	内乡 188	4.250
L252	Zao Yuan Si ZM1763	枣园寺 ZM1763	4.250
L255	Bai Sui Bai ZM1705	白穗白 ZM1705	4.250
L270	Tai Nong 142	泰农 142	4.250
L379	Chuan Mai 44	川麦 44	4.250
L386	Shan Mai 509	陕麦 509	4.250
L400	Xi Nong 3517	西农 3517	4.250
L145	Lun Xuan 987	轮选 987	4.330
L160	Shi Xin 828	石新 828	4.330

L279	Han 6172	邯 6172	4.330
L293	Yang Mai 17 Hao	扬麦 17 号	4.330
L30	Zhong Liang 19	中梁 19	4.330
L332	Lan 21	兰 21	4.330
L365	Fu 936	阜 936	4.330
L470	Dao Tian Pin Zhong ZM1858	稻田品种 ZM1858	4.330
L188	85Zhong 33	85 中 33	4.430
L292	Ping Yuan 50	平原 50	4.430
L368	Dong Mai 17	鄂麦 17	4.430
L399	Jin Mai 47	晋麦 47	4.430
L430	Chuan Mai 49	川麦 49	4.430
L432	Wan Mai 53	皖麦 53	4.430
L113	Wan Mai 33	皖麦 33	4.500
L129	Ji Mai 22	济麦 22	4.500
L198	Zheng Zhou 005	郑州 005	4.500
L377	Xiang Mai 55	襄麦 55	4.500
L392	Xin Dong 38 Hao (0138)	新冬 38 号(0138)	4.500
L414	Shi Xin 618	石新 618	4.500
L449	Jin Mai 50	晋麦 50	4.500
L126	Chuan Mai 24	川麦 24	4.600
L132	Feng Chan 3 Hao	丰产 3 号	4.600
L370	Yu Mai 9 Hao	豫麦 9 号	4.600
L374	Yu Nong 202	豫农 202	4.600
L394	19HRWSN-76	19HRWSN-76	4.600
L84	Zhong Liang 23	中梁 23	4.600
L154	Zhong Mai 415	中麦 415	4.670
L261	Lovrin13	lovrin13	4.670
L367	Ji Mai 26	冀麦 26	4.670
L393	Jing 411	京 411	4.670
L428	Yan Zhan 4110	偃展 4110	4.670
L490	Dong Mai 15	鄂麦 15	4.670
L142	Jin Mai 61	晋麦 61	4.710
L177	Yan Nong 15	烟农 15	4.710
L427	Feng You 6 Hao	丰优 6 号	4.710
L487	Zhong Liang 18	中梁 18	4.710
L489	Ai Feng 3 Hao	矮丰 3 号	4.710
L493	Jing Dong 22	京冬 22	4.710
L439	Zhou Mai 22	周麦 22	4.750
L491	Zhong Nong 28	中农 28	4.750
L53	Gui Nong 16	贵农 16	4.750
L239	Lai Yang Bang ZM1744	莱阳棒 ZM1744	4.763
L338	Long Yuan 994	陇原 994	4.830
L104	Long Jian 196	陇鉴 196	5.000
L107	Zhou Mai 18	周麦 18	5.000

L109	Xin Dong 37 Hao	新冬 37 号	5.000
L127	Su 553	宿 553	5.000
L134	Lu Yuan 502	鲁原 502	5.000
L138	Zhong Yu 5 Hao	中育 5 号	5.000
L144	Ning Dong 11 Hao	宁冬 11 号	5.000
L146	Xi Ke Mai 6 Hao	西科麦 6 号	5.000
L155	Yu Mai 51 (Zhou Mai 11)	豫麦 51(周麦 11)	5.000
L157	Ji Mai 32	冀麦 32	5.000
L158	Wan Mai 47	皖麦 47	5.000
L164	Su Mai 3 Hao	苏麦 3 号	5.000
L168	Jin Mai 67	晋麦 67	5.000
L180	Ji Mai 41	冀麦 41	5.000
L181	Ke Nong 199	科农 199	5.000
L187	Shang Hai 3 Hao	上海 3 号	5.000
L20	Zheng Mai 004	郑麦 004	5.000
L216	Tou Qing Zi ZM1896	透庆子 ZM1896	5.000
L246	klein jabal 1	klein jabal 1	5.000
L25	Chuan Yu 21	川育 21	5.000
L251	Dong Mao Zi ZM1780	二毛子 ZM1780	5.000
L254	Liang Mai ZM1690	亮麦 ZM1690	5.000
L273	You Zi Mai	柚子麦	5.000
L275	Ban Jie Hong Sui ZM1925	半截红穗 ZM1925	5.000
L280	Fan Mai 5 Hao	泛麦 5 号	5.000
L285	Cang Mai 028	沧麦 028	5.000
L287	Chuan Yu 18	川育 18	5.000
L29	Chuan Nong 18	川农 18	5.000
L291	Heng Guan 33	衡观 33	5.000
L304	Chuan Mai 42	川麦 42	5.000
L305	Ning Dong 10 Hao	宁冬 10 号	5.000
L313	Xin Dong 28 Hao	新冬 28 号	5.000
L314	Mian Yang 351-15	绵阳 351-15	5.000
L343	(Ai Gan) Bi Ma 4 Hao	(矮秆)碧蚂 4 号	5.000
L350	Lu Mai 20	鲁麦 20	5.000
L363	Zheng Mai 9023	郑麦 9023	5.000
L366	Ning Mai 17 Hao	宁麦 17 号	5.000
L384	Yu Nong 201	豫农 201	5.000
L387	Chuan Nong 22	川农 22	5.000
L388	Lan 18	兰 18	5.000
L397	Xi Nong 6028	西农 6028	5.000
L398	Guan 35	观 35	5.000
L402	Heng 4338	衡 4338	5.000
L403	Lu Mai 5 Hao	鲁麦 5 号	5.000
L405	Yu Mai 55	豫麦 55	5.000
L408	Xin Mai 9408	新麦 9408	5.000

L410	Jing Dong 17	京冬 17	5.000
L411	Lu Mai 15	鲁麦 15	5.000
L412	Xin Dong 16 Hao	新冬 16 号	5.000
L416	Chuan Mai 107	川麦 107	5.000
L420	Kui Hua 1 Hao	奎花 1 号	5.000
L426	Dong Mai 27	鄂麦 27	5.000
L429	Yan Nong 18	烟农 18	5.000
L434	Yi Nong 18 Hao	伊农 18 号	5.000
L436	Nei Mai 8 Hao	内麦 8 号	5.000
L441	mangnus	mangnus	5.000
L446	Long Jian 294	陇鉴 294	5.000
L488	Lu Mai 23	鲁麦 23	5.000
L6	Liang Mai 4 Hao	良麦 4 号	5.000
L63	Chuan Yu 17	川育 17	5.000
L69	Lan 10	兰 10	5.000
L75	Xi Nong 2611	西农 2611	5.000
L8	Xi Nong 538	西农 538	5.000
L85	Tai Shan 1 Hao	泰山 1 号	5.000
L86	Shi Dong 8 Hao	石冬 8 号	5.000
L46	Ji Mai 19	济麦 19	5.140
L329	Xiao Yan 228	小偃 228	5.168
L317	Yang Mai 158	扬麦 158	5.250
L135	Zhen Mai 6 Hao	镇麦 6 号	5.290
L182	Chuan Mai 39	川麦 39	5.290
L22	Lan 23	兰 23	5.290
L315	Chuan Mai 50	川麦 50	5.290
L395	Lu Mai 8 Hao	鲁麦 8 号	5.290
L41	Ning Mai 16 Hao	宁麦 16 号	5.290
L468	Sha Gou Mang ZM1766	沙沟芒 ZM1766	5.290
L176	Yan 893 Xuan	偃 893 选	5.330
L189	Chang Wu 134	长武 134	5.330
L295	Wei Mai 8 Hao	潍麦 8 号	5.333
L309	Xiao Yan 216	小偃 216	5.333
L272	Hong Tu Tou ZM1707	红秃头 ZM1707	5.380
L208	Yu Nong 416	豫农 416	5.400
L277	Xiao Yan 228	小偃 22	5.400
L307	Chuan Nong 24	川农 24	5.400
L471	Zi Jing Qing ZM1778	紫茎青 ZM1778	5.400
L77	Luo Mai 21	洛麦 21	5.400
L110	Xi Nong 9871	西农 9871	5.500
L184	Ke Nong 213	科农 213	5.500
L209	Huai Mai 18	淮麦 18	5.500
L301	Jin Mai 33	晋麦 33	5.500
L306	Nan Nong 9918	南农 9918	5.500

L361	Gao Cheng 8901	藁城 8901	5.500
L148	Yu Mai 50 (Feng You 5 Hao)	豫麦 50(丰优 5 号)	5.668
L185	Chuan Nong 7 Hao	川农 7 号	5.670
L245	Darius	darius	5.670
L278	Nei Mai 9 Hao	内麦 9 号	5.670
L4	Yan Nong 19	烟农 19	5.670
L97	Dong Mai 352	鄂麦 352	5.670
L50	Lai Yang 4671	莱阳 4671	5.750
L183	Xin Dong 19 Hao	新冬 19 号	5.800
L238	Bai Tang Yang Mai ZM1762	白糖洋麦 ZM1762	5.800
L448	Bei Jing 0045	北京 0045	5.800
L5	Jin Duo 1 Hao	金铎 1 号	5.800
L106	Ning Dong 5 Hao	宁冬 5 号	5.890
L101	Jin Mai 49 (Lin Fen 118)	晋麦 49(临汾 118)	6.000
L102	Han 5316	邯 5316	6.000
L173	Zhong Liang 21	中梁 21	6.000
L191	Xin Dong 32 Hao	新冬 32 号	6.000
L213	Ning Dong 3 Hao	宁冬 3 号	6.000
L26	Chuan Nong 17	川农 17	6.000
L284	Chuan Yu 20	川育 20	6.000
L327	Ai Kang 58	矮抗 58	6.000
L348	Dong Mai	豆麦	6.000
L40	Xin Dong 17 Hao	新冬 17 号	6.000
L431	Mian Za Mai 168	绵杂麦 168	6.000
L474	Bai Mai (San) ZM1687	白麦（三）ZM1687	6.000
L73	Liao Mai 18	聊麦 18	6.000
L74	Gao You 503 (Xiao Yan 503)	高优 503(小偃 503)	6.068
L117	Cang 6003	沧 6003	6.068
L95	Zhen Mai 168	镇麦 168	6.110
L108	Yang Mai 13 Hao	扬麦 13 号	6.140
L125	Xu Mai 26	徐麦 26	6.140
L205	Chuan Nong 27	川农 27	6.140
L37	Yu 356-9	渝 356-9	6.200
L93	Dong Mai 18	鄂麦 18	6.330
L192	Jin Mai 45	晋麦 45	6.330
L199	Lan 27	兰 27	6.330
L203	Dorico	dorico	6.330
L288	Xu Mai 27	徐麦 27	6.330
L297	Long Mai 157 (Ping Liang 44 Hao)	陇麦 157(平凉 44 号)	6.330
L316	Xiao Yan 4 Hao	小偃 4 号	6.330
L396	Bai Gao 38	白高 38	6.330
L49	Zhong Liang 17	中梁 17	6.333
L80	Shan 558	陕 558	6.430

L190	Heng 7228	衡 7228	6.500
L136	Tai Shan 9818 (Da Li)	泰山 9818(大粒)	6.500
L165	Dong Dong 5 Hao	鄂恩 5 号	6.500
L18	Mian Mai 40	绵麦 40	6.500
L212	donski-93	donski-93	6.500
L319	Lan Kao 906	兰考 906	6.500
L390	Jing Dong 8 Hao	京冬 8 号	6.500
L456	Ren Mang Mai ZM1931	人芒麦 ZM1931	6.600
L221	Da You Zi ZM1813	大蚰子 ZM1813	6.668
L89	Rong Mai 2 Hao	蓉麦 2 号	6.710
L473	kitanokaori	kitanokaori	6.750
L120	Liang Xing 99	良星 99	6.750
L55	Yang Mai 9 Hao	扬麦 9 号	6.750
L79	Hui Xian Hong	辉县红	7.000
L103	Xin Mai 9 Hao	新麦 9 号	7.000
L197	Ning Mai 13 Hao	宁麦 13 号	7.000
L206	Lai Zhou 953	莱州 953	7.000
L207	Yang Mai 12	扬麦 12	7.000
L21	Xi Nong 979	西农 979	7.000
L229	Hong Ju Mai ZM1774	红菊麦 ZM1774	7.000
L231	Hong Jing Bian Sui ZM1784	红颈扁穗 ZM1784	7.000
L233	Xin Li Jun ZM1748	心里俊 ZM1748	7.000
L234	Lao Jin Mai ZM1799	老金麦 ZM1799	7.000
L27	Yan You 361	烟优 361	7.000
L274	Shu Mai 375	蜀麦 375	7.000
L28	Xin Dong 24 Hao	新冬 24 号	7.000
L290	Bei Jing 841	北京 841	7.000
L296	Mian Mai 37	绵麦 37	7.000
L298	Chuan Mai 47	川麦 47	7.000
L32	Xi Ke Mai 1 Hao	西科麦 1 号	7.000
L353	Liang Mai 2 Hao	良麦 2 号	7.000
L354	Lan 22	兰 22	7.000
L444	Ke Cheng Mai 2 Hao	科成麦 2 号	7.000
L59	Huang Xian Da Li Ban Mang	黄县大粒半芒	7.000
L7	Ning Dong 13 Hao	宁冬 13 号	7.000
L91	Lan 15	兰 15	7.000
L219	Xiao Hong Mang ZM1907	小红芒 ZM1907	7.058
L196	Xin Dong 31 Hao	新冬 31 号	7.290
L128	Mian Yang 32	绵阳 32	7.330
L210	Xiao Bai Sui ZM1822	小白穗 ZM1822	7.330
L230	sunstate	sunstate	7.400
L459	Zhang Si Huang ZM5525	丈四黄 ZM5525	7.400
L276	Dong Mai ZM4781	短麦 ZM4781	7.500
L61	Shan 229	陕 229	7.670

L96	Chuan Mai 48	川麦 48	7.670
L34	Ji Mai 21	济麦 21	7.880
L105	Zhou Mai 25	周麦 25	8.000
L224	Lai Yang Huang Mai ZM1796	莱阳黄麦 ZM1796	8.000
L299	Lan Kao Ai Zao 8	兰考矮早 8	8.000
L389	Bian Sui Mai	扁穗麦	8.000
L303	Tai Shan 5 Hao	泰山 5 号	8.200
L465	Zi Jie Mang ZM1021	紫秸芒 ZM1021	8.200
L38	Ji 5265	冀 5265	8.250
L310	Shi Mai 12	石麦 12	8.330
L36	Hua Bei 187	华北 187	8.430
L11	Lan 6	兰 6	8.558
L147	Xin Dong 15 Hao	新冬 15 号	8.558
L217	Yu Shi Wa Wa (Yuan) ZM1890	玉石娃娃（原）ZM1890	8.710
L116	Zi Mai 12	淄麦 12	9.000
L118	Shan 253	陕 253	9.000
L119	Ji Mai 30	冀麦 30	9.000
L13	Chuan Mai 52	川麦 52	9.000
L14	Mian Mai 39	绵麦 39	9.000
L149	Shan Nong Fu 63	山农辐 63	9.000
L150	Xi Nong 928	西农 928	9.000
L156	Shi Xin 811	石新 811	9.000
L178	Mian Yang 30	绵阳 30	9.000
L19	Yu Nong 209	豫农 209	9.000
L200	Yang Mai 15 Hao	扬麦 15 号	9.000
L211	Jin Ba Chi ZM1769	金巴齿 ZM1769	9.000
L214	Hong Lou Gu Ding ZM1730	红蝼蛄腚 ZM1730	9.000
L222	Sui Shou ZM1897	穗收 ZM1897	9.000
L223	Qing Jie Hong Tu Tou ZM1831	青秸红秃头 ZM1831	9.000
L225	batjko	batjko	9.000
L226	Da Huang Pi ZM5892	ZM5892 大黄皮	9.000
L23	Zhong You 9507	中优 9507	9.000
L235	Yu Mai ZM1670	玉麦 ZM1670	9.000
L236	Wu Tong Mang Mai ZM1817	梧桐芒麦 ZM1817	9.000
L237	Bo Dong Qing (Lan Yan) ZM1768	脖儿青（兰眼）ZM1768	9.000
L240	San Ba Mai ZM1939	三八麦 ZM1939	9.000
L241	Xiao Bai Mai ZM1721	小白麦 ZM1721	9.000
L243	Zhu Bai Mai ZM1865	贮白麦 ZM1865	9.000
L244	Hong Sui Bai Mai ZM1753	红穗白麦 ZM1753	9.000
L308	Yu Mai 14 You	豫麦 14 优	9.000
L31	Xu Mai 4036 (Xu Mai 31)	徐麦 4036（徐麦 31）	9.000
L318	Xi Ke Mai 3 Hao	西科麦 3 号	9.000
L320	Shan Nong M17	山农 M17	9.000

L321	Zhong Mai 175	中麦 175	9.000
L324	Xu Zhou 23	徐州 23	9.000
L325	Yu Mai 1 Hao	玉脉 1 号	9.000
L328	Zhong Liang 30	中梁 30	9.000
L33	Lan 24	兰 24	9.000
L330	Yu Mai 57	豫麦 57	9.000
L331	Zhong Liang 27	中梁 27	9.000
L334	Zhong Yu 9 Hao	中育 9 号	9.000
L335	Yu Mai 7 Hao	豫麦 7 号	9.000
L337	Lan 20	兰 20	9.000
L339	Yang Mai 11 Hao	扬麦 11 号	9.000
L340	Hua Pei 5 Hao	花培 5 号	9.000
L341	An Nong 0305	安农 0305	9.000
L346	Dong Dong 1 Hao	鄂恩 1 号	9.000
L349	Chuan Nong 16	川农 16	9.000
L351	Shi 4185	石 4185	9.000
L358	Yu Mai 2 Hao	豫麦 2 号	9.000
L359	He Nong 6049	河农 6049	9.000
L360	Dong Mai 14	鄂麦 14	9.000
L39	Shan Mai 159	陕麦 159	9.000
L391	Zhou Mai 32	周麦 32	9.000
L406	Yu Mai 41	豫麦 41	9.000
L422	Han Mai 14	邯麦 14	9.000
L43	Lu Mai 16	鲁麦 16	9.000
L44	Feng You 68	丰优 68	9.000
L45	Kui Dong Si Hao	奎冬四号	9.000
L451	Yan 2415	烟 2415	9.000
L458	Jin Mai ZM1821	进麦 ZM1821	9.000
L475	Hong Tang Liang Mai ZM1761	红糖良麦 ZM1761	9.000
L478	Zhong Guo Chun	中国春	9.000
L52	He Nong 827	河农 827	9.000
L56	Bi Ma 1 Hao	碧蚂 1 号	9.000
L57	Wen Nong 14	汶农 14	9.000
L58	Zheng Yin 1 Hao	郑引 1 号	9.000
L60	Nong Da 212	农大 212	9.000
L65	Yi Mai 8 Hao	宜麦 8 号	9.000
L67	Xin Dong 23 Hao	新冬 23 号	9.000
L68	Xu Zhou 25	徐州 25	9.000
L70	Ning Mai 8 Hao	宁麦 8 号	9.000
L72	Lu Mai 3 Hao	鲁麦 3 号	9.000
L78	Nei Xiang 182 (Yu Mai 17 Hao)	内乡 182(豫麦 17 号)	9.000
L81	Shi Xin 733	石新 733	9.000
L82	Xi Nong 88	西农 88	9.000

L83	Zhong Liang 25	中梁 25	9.000
L87	Yang Mai 4 Hao	扬麦 4 号	9.000
L88	Chuan Yu 19	川育 19	9.000
L9	Ning Chun 10 Hao	宁春 10 号	9.000
L94	Xin Dong 22 Hao	新冬 22 号	9.000

Table S2. Pearson correlations of *Fusarium* crown rot reactions in three trials and BLUP

Systematic repeats	Rep_1	Rep_2	Rep_3	BLUP
**Rep_1**	1			
**Rep_2**	0.82	1		
**Rep_3**	0.87	0.93	1	
**BLUP**	0.94	0.96	0.97	1

Table S3. Haplotype analysis on 12 SNPs within the *Qfcr.hebau-3BL* physical interval.

**Name of germplasm**	**Resistance level**	**Disease Index**	**Haplotype**	**AX-109558127**	**AX-111096287**	**AX-109494780**	**AX-109535066**	**AX-108760361**	**AX-109294391**	**AX-111242526**	**AX-110998977**	**AX-109470333**	**AX-111645949**	**AX-111121474**	**AX-111562784**
Lumai14	R	1.86	*Qfcr(3BL)*	C	A	A	A	A	T	A	G	C	C	A	T
Ningmai 15	R	1.00	*Qfcr(3BL)*	C	A	A	A	A	T	A	G	C	C	A	T
Zhengmai 366	R	1.45	*Qfcr(3BL)*	C	A	A	A	A	T	A	G	C	C	A	T
Langyan 43	R	2.25	*Qfcr(3BL)*	C	A	A	A	A	T	A	G	C	C	A	T
WGRC10/3/KS93U69 sib/TA2455//KS93U69/4/JAGGER	R	1.84	*Qfcr(3BL)*	C	A	A	A	A	T	A	G	C	C	A	T
Yumai 57	S	9.00	*Qfcr(-)*	T	G	G	G	C	C	A	G	C	C	A	T
Yangmai 11	S	9.00	*Qfcr(-)*	T	G	G	G	C	C	A	G	C	C	A	T
Shi 4185	S	9.00	*Qfcr(-)*	T	G	G	G	C	C	A	G	C	C	A	T
Emai 14	S	9.00	*Qfcr(-)*	T	G	G	G	C	C	A	G	C	C	A	T
Jinmai ZM1821	S	9.00	*Qfcr(-)*	T	G	G	G	C	C	A	G	C	C	A	T

Table S4. CAPS/dCAPS markers were designed based on the SNPs associated with *Qfcr.hebau-3BL*.

**Markers Name**	**Primer sequence (5'-3')**	**Marker type**	**SNP location/variation**	**Expected size (bp)** [a]	**Annealing temperature (℃)**	**Restriction enzyme**
3B-AX-110998977	F: TTTTTACCAAGTGTCTGCATTATT<br>R: CTTGCCTAGCTATCATATAATTCAC	CAPs	chr3B:494812710<br>G/A	182	55.5	EcoR I
3B-AX-109294391	F: TACAATGCCGGTTAAGTTGATGAGC<br>R: AACCACATTGCCAAGTTATTGAAGA	dCAPs	chr3B:494529843<br>G/A	224	63.1	Hha I
3B-AX-110960287	F: GGATAGCACCCTCGTCGG<br>R: TTTGAAGATGTTGACTCCATATGTT	dCAPs	chr3B:494078089<br>C/T	222	58.0	Hph I

[a] the expected PCR product size before digestion

Table S5. Primers used in this study.

Primer usage	Primer name	Primer sequences 5' to 3'	PCR product	Accession
pBIN-GFP	TaSTK1.1-KpnI-F	ggtaccATGGACGAGGAGGATTACTCG	1158bp	*TraesCS3B02G307700.1*
	TaSTK1.1-ST-BamHI-R:	ggatccTCAACGAACCACCAGAAAGGAACT		
	TaSTK1.2-KpnI-F	ggtaccATGGACGAGGAGGATTACT	1812 bp	*TraesCS3B02G307700.2*
	TaSTK1.2-ST-BamHI-R:	ggatccTTACAGAAGTTTTGGTGGTGG		
